# Usefulness of the combined orthodontic rubber band and clip method for gastric endoscopic submucosal dissection

**DOI:** 10.1186/s12876-022-02606-1

**Published:** 2022-12-17

**Authors:** Dazhou Li, Linfu Zheng, Zewen Zhang, Longping Chen, Chuanshen Jiang, Rong Wang, Jiahong Lin, Yiwen Lu, Yang Bai, Wen Wang

**Affiliations:** 1 Department of Gastroenterology, The 900th Hospital of PLA, Fuzhou General Clinical Medical College, Fujian Medical University, Fuzhou, 350025 China; 2 Guangdong Provincial Key Laboratory of Gastroenterology, Department of Gastroenterology, Institute of Gastroenterology of Guangdong Province, Nanfang Hospital, Southern Medical University, Guangzhou, 510515 China; 3 Department of Gastroenterology, 900th Hospital of People’s Liberation Army, Fuzhou, 350025 China; 4 Department of Gastroenterology, Oriental Hospital Affiliated to Xiamen University, Fuzhou, 350025 China

**Keywords:** Orthodontic rubber band, Endoscopic mucosal dissection, Traction method

## Abstract

**Background and aims:**

Effective traction is an important prerequisite for successful endoscopic submucosal dissection (ESD). The combined orthodontic rubber band (ORB) and clip method was effective in colorectal cancer ESD. To date, the method was not reported in gastric ESD. This study aimed to investigate its efficacy and safety for gastric neoplasms ESD.

**Methods:**

We retrospectively analyzed data of 118 patients with gastric neoplasms treated by ESD from November 2020 to April 2022, 43 by ORB-ESD and 75 by the conventional ESD. The primary outcome measure was the ESD procedure time. Clinical data on efficacy and safety were also collected and analyzed. Propensity score matching (PSM) matched the patients in both groups.

**Results:**

PSM successfully matched 31 pairs of patients. The ORB-ESD operation time was shorter (median [interquartile range], 35 [30–48] vs. 49 [40–70] min, *P* < 0.001) and dissection speed was higher (median [interquartile range], 22.6 [14.4–29.3] vs. 13.5 [9.6–17.9] mm^2^/min, *P* < 0.001) than in the conventional ESD. The groups were similar in muscular injury rate, frequency and time of use of thermal hemostatic forceps, postoperative adverse events, en bloc resection, and R0 resection rate (*P* > 0.05).

**Conclusions:**

Compared to the conventional ESD, ORB-ESD significantly reduced the procedure time and increased the dissection speed, proving beneficial to gastric ESD.

## Introduction

Endoscopic submucosal dissection (ESD), developed from the endoscopic mucosal resection technique, has become an effective new method to treat early gastric cancer and precancerous lesions because it can completely resect the lesion and reduce the local recurrence rate [1]. However, ESD is more difficult and time-consuming than endoscopic mucosal resection and has a higher rate of adverse events such as bleeding and perforation [2–4]. Therefore, endoscopists are presently highly concerned with finding ways to improve the efficiency of ESD and reduce its adverse events rate.

It has been reported that traction-assisted methods provide endoscopists with a better field of view of the submucosa, shortening the ESD duration and reducing its adverse events rate [5]. Therefore, various ESD traction methods continued to emerge in recent years [6], all showing advantages and disadvantages. Our previous report showed that the combined orthodontic rubber band (ORB) and clip traction-assisted ESD achieved good results in colorectal tumors [7]. ORBs are cheap, simple to operate, safe, and effective in the ESD procedure. No previous study reported this combined ORB and clip method in gastric ESD. Therefore, this study compared and analyzed patients with gastric neoplasms treated by ORB-ESD and the conventional ESD at our hospital, assessing the efficacy and safety of ORB-ESD for gastric neoplasms.

## Materials and methods

This study included patients with superficial gastric neoplasms (early gastric cancer and precancerous lesions or gastric adenomas with a high risk of cancer) who underwent ESD at the 900^th^ Hospital of the PLA from November 2020 to April 2022. All patients were scheduled to undergo ESD following the Chinese consensus on early gastric cancer treatment [8]. ESD was recommended even if a submucosal invasion was suspected when the patient strongly preferred this procedure rather than surgery. Exclusion criteria were: (1) patients in whom other traction methods such as dental floss or endoscopic submucosal tunnel dissection (ESTD) were used; (2) patients who underwent gastrectomy (ESD is difficult in such patients) [9]. If multiple gastric lesions underwent ESD, only lesion dissected first was included in this study. Patients taking anticoagulants were asked to stop taking them 5–7 days before the ESD procedure [10].

The ethics committee of the 900th Hospital of the PLA approved this retrospective study (institutional ID 2022-012). The ESD procedures were performed after the patients gave their informed consent.

### ESD equipment

**Fig.1:**
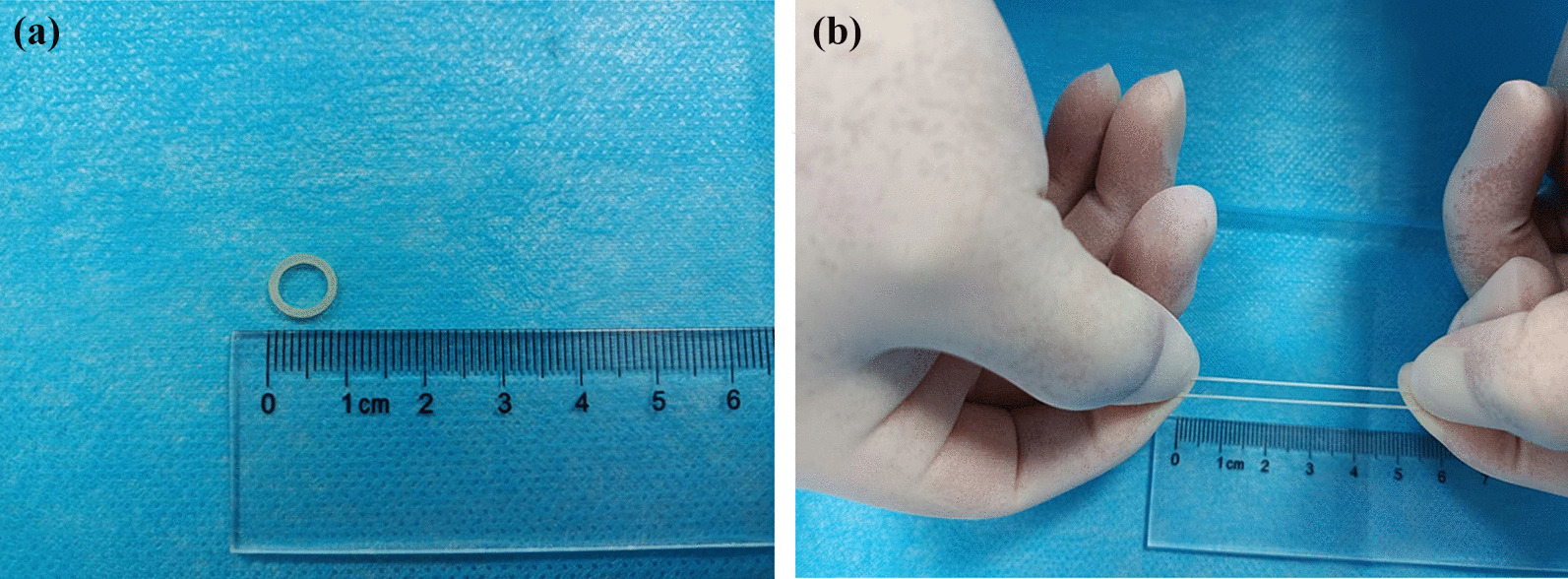
Photograph of an orthodontic rubber band (ORB). **A** ORB (inner diameter, 6.5 mm); **B** ORB with excellent elasticity

All ESD procedures were performed with a single-channel endoscope with a water jet system (GIF-Q260J; Olympus Co., Tokyo, Japan), a transparent cap, and an electrosurgical generator (VIO200S; Elektromedizin Gmbh, Tubingen, Germany). We used 1:100,000 sodium adrenaline hyaluronate and an injection needle (nm-4U; Olympus Co.) for submucosal injection. A Dual knife (KD-650L, Olympus Co.) or golden knife (MK-T-2-195, Micro-tech (Nanjing) Co., Nanjing, China) was used for marking, incision, and submucosal dissection. Carbon dioxide was used for gas injection. Other equipment included opening-and-closing clip (Micro-tech (Nanjing) Co., Nanjing, China), ORB (inner diameter, 6.5 mm, (1/4”), 3.5 OZ.) (Fig. 1), and hemostatic forceps (Coagrasper, Olympus Co.).

### The ESD procedure

**Fig. 2:**
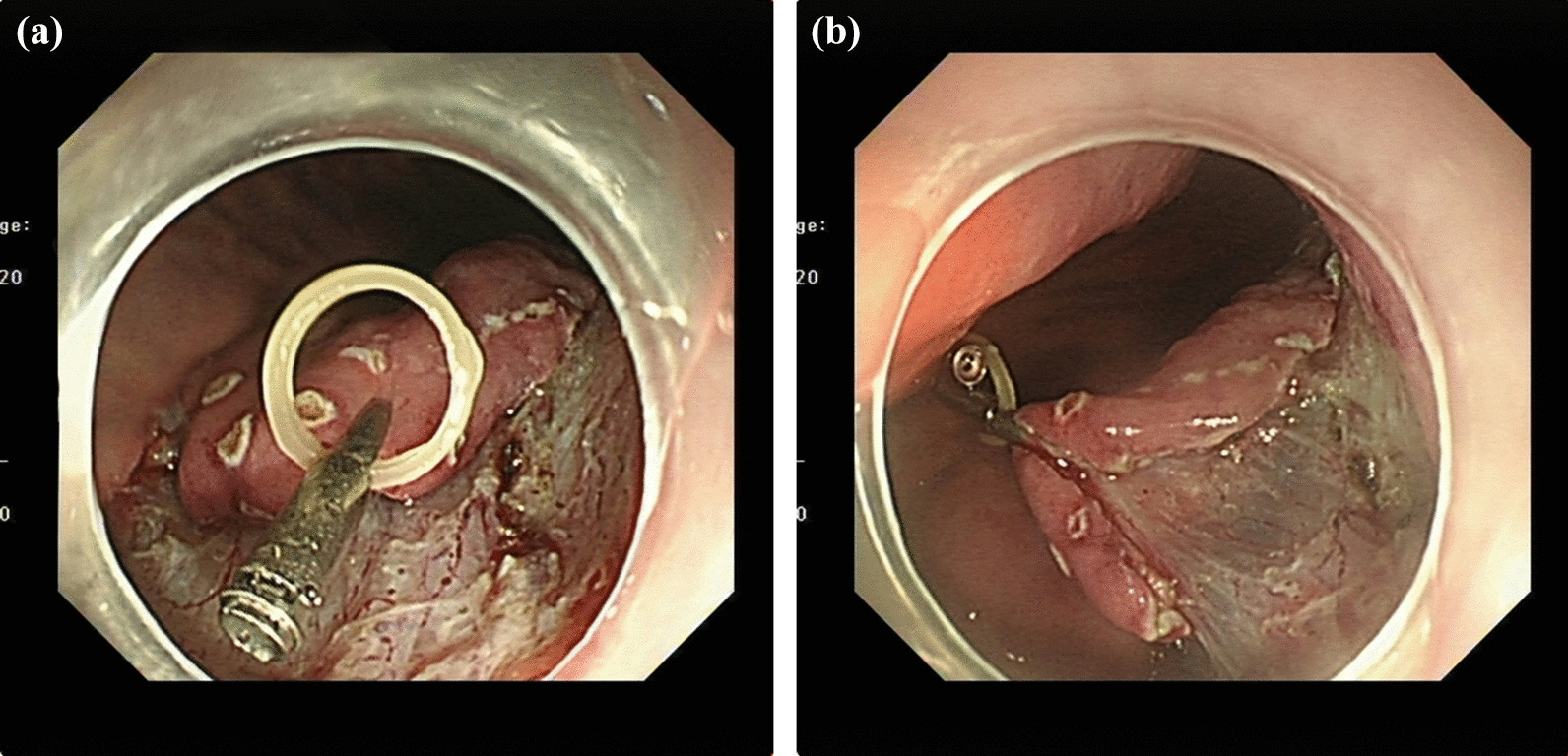
The procedure of ORB-ESD. **A** The incision mucosa was attached with an ORB combined clip; **B** The clip was anchored on the opposite side of the lesion to form effective traction, and the submucosa was well exposed

**Fig. 3:**
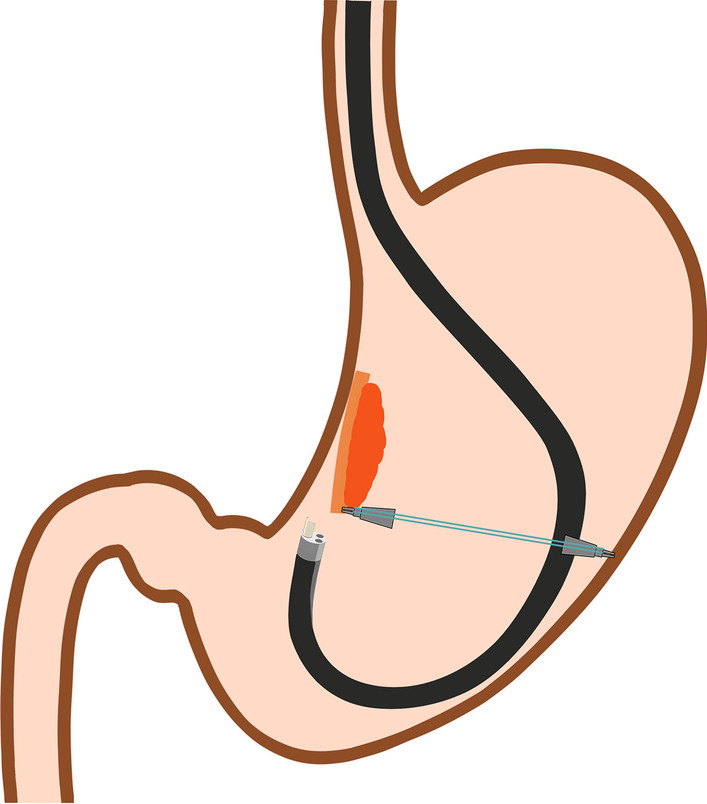
Schematic illustration of retroflexed endoscopic position for submucosal dissection

The conventional ESD process was as follows: a Dual knife or golden knife was used to open a 5-mm line mark next to the lesion. Subsequently, we performed submucosal injection and lifting, followed by an incision around the lesion and dissection using the Dual knife or golden knife. ORB traction was selected according to the specific conditions of gastric lesions. In the ORB-ESD group (Fig. 2), a clip holding the ORB was inserted into the gastric cavity through the endoscope working channel and clamped to the lesion mucosa after performing an incision around it. Subsequently, another clip was passed through the working channel into the stomach and attached the ORB to the contralateral normal mucosa. The lesion was then dissected. After complete resection of the lesion, the ORB traction device fixed on gastric mucosa and the specimen were removed with a snare. If we need to the retroflexed endoscopic position for submucosal dissection, set the direction of traction in advance, and try to turn the endoscope over from the greater curvature in the retroflexed process, so that the operation is relatively not easy to encounter the traction device (Fig. 3).

### Outcome measures

The primary outcome measure was the ESD procedure duration. The secondary outcome measures included the dissection speed, muscular injury rate, frequency and time of thermal hemostatic forceps use, and the rates of en bloc resection, R0 resection, bleeding, and perforation. In the ORB-ESD group, we also counted the times the ORB was attached to the lesion and whether the ORB traction device affected the specimen integrity.

The procedure time was defined as the time from submucosal injection to complete lesion resection. This interval included the time for inserting and attaching the ORB system. Time was recorded in minutes. The dissection speed was defined as the specimen resected area divided by the procedure duration (mm^2^/min). The specimen area was measured by multiplying half the length by half the width by 3.14 [11].

Muscular injury was defined as the damage caused to the muscle layer during electrosurgery. The frequency of hemostatic forceps use refers to the number of active bleeding requiring the use of the hemostatic forceps. Hemostasis time was defined as the time interval from the appearance of the hemostatic forceps on the monitor to the completion of hemostasis. The total time dedicated to hemostasis during the procedure was calculated [12]. En bloc resection means that the lesion was removed by endoscopy, and an entire sample was obtained. R0 resection means that negative margins were achieved in the horizontal and vertical directions. Bleeding included immediate and delayed bleeding. Immediate bleeding was defined as bleeding that occurred during the ESD procedure and required endoscopic hemostasis. Delayed bleeding was defined as hematochezia within 30 days after surgery and a decrease in hemoglobin by 20 g/L, requiring blood transfusion or endoscopic or surgical intervention. Perforation was defined as the discovery of abdominal fat or intraperitoneal space during the ESD procedure or the presence of free gas in the abdominal cavity viewed on postsurgical radiographs or CT scans.

### Propensity score matching (PSM)

Previous studies reported that lesions in the greater curvature and upper 2/3 of the stomach [13], tumor size [14], ulcer scarring presence [15], and the operator level [16] (trainee with fewer than 40 cases or an expert with over 40 cases of gastric ESD) affected the gastric ESD procedure. Therefore, we used PSM to adjust sampling bias and reduce potential confounding factors. We set the caliper value to 0.2 (nearest neighbor matching ties no replacement) for a 1:1 match to select patients.

### Statistical analysis

Analysis was performed using IBM SPSS Statistics for Windows, Version 22.0 (IBM Corp., Armonk, NY, USA). Categorical variables were compared using the chi-squared or Fisher's exact test (e.g., R0 resection rate and surgical adverse events). Continuous variables were compared using the Student’s *t* test or Mann–Whitney *U* test (e.g., procedure time, dissection speed, and lesion size). Continuous variables are expressed as mean (standard deviation; SD) or median (interquartile range), while categorical variables are expressed as counts and percentages. Differences with *P* < 0.05 were considered statistically significant.

## Results

**Fig. 4:**
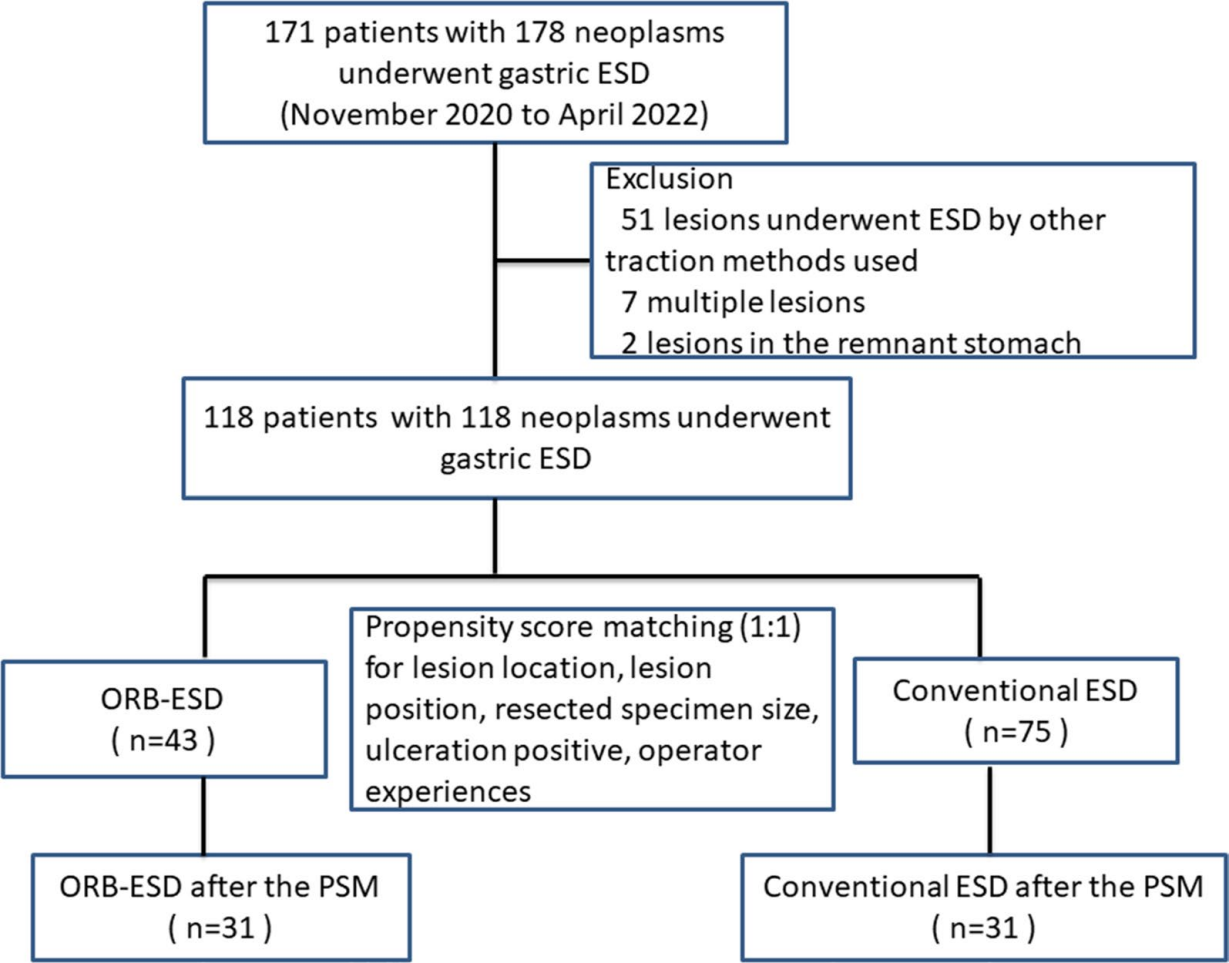
Flowchart of this study design

From November 2020 to April 2022, 171 patients with 178 gastric epithelial neoplasms underwent ESD. After excluding 51 patients (51 lesions) who underwent ESD with dental floss traction or ESTD, two lesions (two patients) in remnant stomachs, and seven patients with multiple lesions, the study included 118 lesions in 118 patients, 43 ORB-ESD and 75 conventional ESD (Fig. 4). The median specimen size in the ORB-ESD group was larger than in the conventional ESD group [40.0 (33.5–48.0) vs. 30.0 (23.0–38.0) mm, *P* < 0.001], while the groups were similar in other baseline characteristics (Table 1). Table 2 shows the results for the two groups before PSM. The median operation time in the ORB-ESD group was insignificantly shorter than in the conventional ESD group [40 (30, 57.5) vs. 44 (35, 61) min, *P* = 0.15], while its median dissection speed was significantly faster [22.6 (16.4, 30.4), vs. 12.6 (7.8, 17.1) mm^2^/min, *P* < 0.001].

**Table 1:** Baseline characteristics of patients who underwent gastric ESD before propensity score matching (PSM)

Variable	ORB-ESD (*n* = 43)	Conventional ESD (*n* = 75)	*P* value
Age, y, (IQR)	67 (60–71)	64 (54–72)	0.161^W^
Sex, *n* (%)			0.438^C^
Male	29 (67)	45 (60)	
Female	14 (33)	30 (40)	
Tumor size, mm (IQR)	20.0 (15.0–30.0)	15.0 (10.0–20.0)	0.001^W^
Specimen size, mm (IQR)	40.0 (33.5–48.0)	30.0 (23.0–38.0)	0.001^W^
Lesion location, *n* (*%*)			0.410^F^
Upper	22 (51)	33 (44)	
Middle	6 (14)	7 (9)	
Lower	15 (35)	35 (47)	
Lesion position, *n* (*%*)			0.108^C^
Greater curvature	4 (9)	19 (25)	
Lesser curvature	14 (33)	18 (24)	
Anterior wall	4 (9)	11 (15)	
Posterior wall	21 (49)	27 (36)	
Macroscopic type, *n* (%)			0.103^C^
Elevated (0-I, 0-IIa)	14 (33)	39 (52)	
Depressed (0-IIb, 0-IIc, 0-III)	19 (44)	26 (35)	
Mixed (0-lla + llc, 0-llc + lla)	10 (23)	10 (13)	
Ulceration positive, *n* (%)	6 (14)	3 (4)	0.071^F^
Operator experiences			0.072^C^
Expert	28 (65)	36 (48)	
Trainee	15 (35)	39 (52)	

*ESD* endoscopic submucosal dissection, *ORB* orthodontic rubber band, *IQR* interquartile range

^W^Mann–Whitney *U* test

^C^Chi-squared test

^F^Fisher’s exact test

**Table 2:** Clinical outcomes of gastric ESD before propensity score matching (PSM)

Variable	ORB-ESD (*n* = 43)	Conventional ESD (*n* = 75)	*P* value
Procedure time, min, (IQR)	40 (30.0–57.5)	44.0 (35.0–61.0)	0.150^W^
Median dissection speed, mm^2^/min (IQR)	22.6 (16.4–30.4)	12.6 (7.8–17.1)	< 0.001^W^
Muscular injury, *n* (%)			0.323^F^
Yes	2 (5)	8 (11)	
No	41 (95)	67 (89)	
Hemostasis frequency			0.259^W^
Mean (SD)	2.35 (2.52)	1.85 (2.23)	
Median (range)	2 (0.0–3.5)	2 (0.0–3.0)	
Time to hemostasis, min			0.311^W^
Mean (SD)	2.47 (2.66)	2.36 (3.22)	
Median (range)	2 (0–4)	0 (0–5)	
ORB-related factors			
Mean ORB attachment time, min (SD)	2.39 (1.67)	NA	NA
ORB clip slip-off, *n* (%)	2 (5)	NA	NA
ORB clip-related damage to specimen, *n* (%)	0 (0)	NA	NA
Successful removal of anchor clip, *n* (%)	43 (100)	NA	NA
Complete resection, *n* (%)	43 (100)	75 (100)	1.000^F^
R0 resection, *n* (%)	41 (95.3)	74 (98.7)	0.553^F^
Horizontal margin positive, *n* (%)	42 (97.7)	75 (100)	0.364^F^
Vertical margin positive, *n* (%)	42 (97.7)	74 (98.7)	1.000^F^
Delayed bleeding, *n* (%)	1 (2.3)	3 (4.0)	1.000^F^
Perforation, *n* (%)	0 (0)	0 (0)	NA
Tumor depth, *n* (%)			0.898^F^
pT1a	39 (91)	66 (88)	
pT1b1	1 (2)	4 (5)	
pT1b2	3 (7)	5 (7)	

*ESD* endoscopic submucosal dissection, *ORB* orthodontic rubber band, *NA* not applicable, *IQR* interquartile range; SD, standard deviation

pT1a, intramucosal cancer; pT1b1, submucosal invasive cancer with invasion depth ≤ 500 µm; pT1b2, submucosal invasive cancer with invasion depth > 500 µm

^W^Mann–Whitney *U* test

^F^Fisher’s exact test

The average time for ORB placement and completion of traction was 2.39 ± 1.67 min. Two patients in the ORB-ESD group lost the clip during the procedure; clips were re-inserted to complete traction. All clips were successfully removed in this group, and no bleeding event due to the removal of the ORB clips or damage to the specimens caused by the ORB device was noted (Table 2).

Table 3 shows the patient characteristics and results after PSM. The groups were similar in specimen size, lesion location, lesion position, macroscopic type, ulceration positive, and operator experiences. The matched groups were similar in muscular injury rate, frequency and time of hemostasis, complete resection, R0 resection rate, postoperative adverse events, and postoperative pathology (*P* > 0.05 for all). However, compared to the conventional ESD group, the ORB-ESD group had a significantly shorter median operation time (35 [30–48] vs. 49 [40–70] min, *P* < 0.001) and faster dissection speed (22.6 [14.4–29.3] vs. 13.5 [9.6–17.9] mm^2^/min, *P* < 0.001).

**Table 3:** Matching factors and clinical outcomes after propensity score matching (PSM)

Variable	ORB-ESD (*n* = 31)	Conventional ESD (*n* = 31)	*P* value
Tumor size, mm (IQR)	20.0 (12.0–25.0)	18.0 (9.0–25.0)	0.420^W^
Specimen size, mm, mean (SD)	37.8 (11.5)	35.6 (11.4)	0.454^T^
Lesion location, *n* (%)			1.000^F^
Upper	14 (45)	15 (48)	
Middle	3 (10)	3 (10)	
Lower	14 (45)	13 (42)	
Lesion position, *n* (%)			0.379^F^
Greater curvature	4 (13)	8 (26)	
Lesser curvature	10 (32)	11 (36)	
Anterior wall	2 (7)	3 (10)	
Posterior wall	15 (48)	9 (28)	
Ulceration positive, *n* (%)	3 (9.7%)	3 (9.7%)	0.612^F^
Operator experiences, n (%)			0.596^C^
Expert	19 (61)	21 (68)	
Trainee	12 (39)	10 (32)	
Procedure time, min, (IQR)	35 (30–48)	49 (40–70)	< 0.001^W^
Median dissection speed, mm^2^/min (IQR)	22.6 (14.4–29.3)	13.5 (9.6–17.9)	< 0.001^W^
Muscular injury, n (%)	0 (0.0)	4 (12.9)	0.113^F^
Frequency of hemostasis			0.262^W^
Mean (SD)	2.22 (2.56)	2.81 (2.39)	
Median (IQR)	2 (0–4)	3 (0–4)	
Time to hemostasis, min			0.112^W^
Mean (SD)	2.05 (2.26)	3.55 (3.58)	
Median (IQR)	2 (0–4)	3 (0–6)	
Complete resection, *n* (%)	31 (100)	31 (100)	NA
R0 resection, *n* (%)	31 (100)	30 (97)	1.000^F^
Horizontal margin positive, *n* (%)	31 (100)	31 (100)	NA
Vertical margin positive, *n* (%)	31 (100)	30 (97)	1.000^F^
Delayed bleeding, *n* (%)	1 (3.2)	2 (6.5)	1.000^F^
Perforation, *n* (%)	0 (0)	0 (0)	NA
Tumor depth, *n* (%)			1.000^F^
pT1a	28 (90)	27 (87)	
pT1b1	1 (3.0)	2 (6.5)	
pT1b2	2 (7.0)	2 (6.5)	

pT1a, intramucosal cancer; pT1b1, submucosal invasive cancer with invasion depth ≤ 500 µm; pT1b2, submucosal invasive cancer with invasion depth > 500 µm

*ESD* endoscopic submucosal dissection, *ORB* orthodontic rubber band, *NA* not applicable, *IQR* interquartile range; SD, standard deviation

^W^Mann–Whitney *U* test

^C^Chi-squared test

^F^Fisher’s exact test

^T^Student’s *t* test

Table 4 shows subgroup analysis results. When the lesion was ≤ 2 cm, in the middle or upper stomach, or anywhere other than the greater curvature, the median surgical time was shorter, and the median dissection speed was faster in the ORB-ESD group than in the conventional ESD group.

**Table 4:** Subgroup analysis of procedure time and dissection speed stratified by the lesion size, location, and position

Variable	ORB-ESD (*n* = 31)	Conventional ESD (*n* = 31)	*P* value
*Tumor size, mm*			
≤ 2 cm	*n* = 19	*n* = 19	
Median Procedure time, min (IQR)	34.0 (23.0–39.0)	46.0 (38.0–69.0)	< 0.001^M^
Median dissection speed, mm^2^/min (IQR)	21.3 (12.1–29.3)	10.9 (6.6–14.0)	0.002^M^
> 2 cm	*n* = 12	*n* = 12	
Median Procedure time, min (IQR)	49.0 (32.5–59.5)	61.0 (44.8–107.0)	0.112^M^
Median dissection speed, mm^2^/min (IQR)	23.5 (14.6–30.6)	15.9 (12.9–20.9)	0.065^M^
*Tumor location*			
Upper/middle	*n* = 17	*n* = 18	
Median Procedure time, min (IQR)	35.0 (30.0–42.5)	52.0 (39.8–76.3)	0.003^M^
Median dissection speed, mm^2^/min (IQR)	24.4 (17.9–33.4)	10.9 (6.5–14.6)	< 0.001^M^
Lower	*n* = 14	*n* = 13	
Median Procedure time, min (IQR)	37.0 (22.3–52.0)	47.0 (39.5–69.0)	0.065^M^
Dissection speed, mm^2^/min, mean (SD)	19.2 (10.3)	16.7 (5.6)	0.436^T^
Greater curvature	*n* = 4	*n* = 7	
Procedure time, min, mean (SD)	31.8 (21.1)	57.9 (30.2)	0.165^T^
Dissection speed, mm^2^/min, mean (SD)	15.3 (9.7)	16.4 (8.4)	0.853^T^
Except greater curvature	*n* = 27	*n* = 24	
Median Procedure time, min (IQR)	39.0 (30.0–48.0)	49.5 (40.3–70.8)	0.001^F^
Median dissection speed, mm^2^/min (IQR)	22.6 (14.6–31.4)	12.8 (8.9–15.9)	< 0.001^M^

*ESD* endoscopic submucosal dissection, *ORB* orthodontic rubber band, *IQR* interquartile range, *SD* standard deviation

^M^Mann–Whitney *U* test

^F^Fisher’s exact test

^T^Student’s *t* test

## Discussion

As with any other surgery, the surgical field of view is critical in ESD [17]. Poor submucosal exposure can greatly increase the operation difficulty. Uncertain dissection could lead to adverse events such as bleeding and perforation. By performing traction, the submucosa can be well exposed, achieving a better visual field and improved dissection efficiency. Furthermore, blood vessel branches can be detected, and electrocoagulation be timely applied to reduce the occurrence of bleeding and perforation. Therefore, traction technologies for gastric ESD are constantly emerging. These include double-endoscope endoscopic submucosal dissection [18, 19], ESTD, [17, 20] spring‑and‑loop with clip (S–O clip) traction [21, 22], and dental floss clip traction [12, 23]. Each of these traction technologies has its limitations. For example, two endoscopists should participate in the double-endoscope endoscopic submucosal dissection procedure. ESTD is difficult for trainees to master. The S–O traction technique requires an S–O clip, which is relatively expensive and available only in some endoscopy centers. Therefore, besides dental floss clip traction, these traction methods were not widely used in gastric ESD.

To place the dental floss traction, one needs to exit the endoscope and install the traction device. The traction is in a single direction, and the friction between dental floss and the endoscope might sometimes tear the dental floss or interfere with the ESD procedure. The ORB is commonly used in orthodontics. It comes in various diameters, has good elasticity, is cheap, and can be passed through the endoscope working channel. We showed in our previous study that the ESD procedure could be shortened by using ORB with a clip to assist ESD of colorectal tumors [7]. Therefore, we hypothesized that the use of ORB to assist gastric ESD could improve its efficiency.

Our results showed that the procedure time in the ORB-ESD group was insignificantly shorter than in the conventional ESD group. This could be due to the larger specimen size in the ORB-ESD group than in the conventional ESD group. After matching the groups to reduce the influence of the specimen size, lesion location, ulceration, and operator experience, the procedure time in the ORB-ESD group was significantly shorter than in the conventional ESD group (35 vs. 49 min, *P* < 0.001). Furthermore, the dissection speed in the ORB-ESD group was faster than in the conventional ESD group both before and after PSM (*P* < 0.001). The possible reason for the higher speed could be the tension maintained throughout the ORB-assisted ESD procedure, exposing sufficient submucosa, reducing the operation difficulty, and speeding dissection.

Goto et al. reported that a tumor size greater than 20 mm was a predictor of an ESD surgery longer than 120 min [24]. Our subgroup analysis showed that when the tumor size exceeded 20 mm, the ESD procedure duration and dissection speed were insignificantly better in the ORB-ESD group than in the conventional ESD group. This difference could be because the traction force gradually decreases when dissecting large-area lesions during the ESD process, resulting in a decreased exposure of the submucosa. We have encountered similar situations in the colorectal ORB-ESD. Our experience suggests that placing a clip to clamp the rubber band to the opposite side of the lesion can reestablish effective traction [7]. Therefore, the ESD procedure may be shortened by re-inserting a new ORB for traction when dissecting lesions larger than 20 mm.

It was reported that tumors in the middle and upper stomach were more difficult to manage with ESD, a factor affecting gastric ESD [14, 25]. Our subgroup analysis showed that, compared to the conventional ESD group, the ORB-ESD group had a shorter procedure time (35.0 vs. 52.0 min, *P* = 0.003) and faster dissection speed (24.4 vs. 10.9 mm^2^/min, *P* < 0.001) when the lesion was in the middle or upper stomach. Therefore, we suggest using ORB-assisted ESD to improve the ESD efficiency when lesions are in the upper 2/3 of the stomach. However, when the lesion is in the lower 1/3 of the stomach, especially in the antrum, the submucosa is well exposed even without traction, the endoscope can be well manipulated, and the operation is technically easy. For this reason, our data suggest that the ORB-ESD and conventional ESD groups had similar procedure times and dissection speeds when the lesion was in the lower 1/3 of the stomach (*P* > 0.05), consistent with the report by Nagata [22]. Furthermore, our subgroup analysis suggested that the procedure time and dissection speed were similar in both groups when the lesion was in the greater curvature of the stomach, possibly reflecting the small sample size.

The reported primary adverse events during gastric ESD included bleeding and perforation. The incidence of delayed bleeding was 4.1–8.5% [26–29] and that of perforation was 0.0–2.7% [30, 31]. In our study, the incidences of delayed bleeding in the ORB-ESD and conventional ESD groups were 2.3 and 4.0%, respectively. No perforation occurred in either group, consistent with previous reports [26–31]. Yoshida et al. reported a clip slip-off rate of 13.2% when using dental floss and clip traction [12]. Nagata reported that slip-off rate of 3.9% during S–O clip-assisted gastric ESD [22]. In our study, ORB clips slipped off due to over-inflation in two patients, with an incidence of 5%. Both cases occurred when a trainee performed the procedure. Therefore, it is suggested that trainees performing ORB-ESD should pay attention to avoid clip slip-off caused by excessive insufflation.

This study had some limitations. First, it was a single-center retrospective study. However, we maintain a prospective, continuous database since we started using ORB-assisted ESD, and PSM was used to reduce the influence of confounding factors. Second, while five ORB inner diameters were available (3.16, 4.70, 6.35, 7.90, and 9.50 mm), we used only the 6.35-mm ORB. Further research is needed to determine whether larger-diameter ORB can produce a better traction effect for large neoplasms. Thirdly, for lesions that develop in anatomically narrow areas, such as those near the gastroesophageal junction or pyloric ring, ORB traction may not work well at times. Finally, the sample size in this study was relatively small. The effectiveness of the ORB-assisted ESD procedure could be reconfirmed through future multi-center prospective large-sample studies.

In conclusion, this study showed that using ORB to assist gastric ESD can shorten the procedure time and improve the dissection speed. ORBs are easy to obtain and operate. This technique has the potential to become a simple, safe, and reliable gastric ESD traction method widely used in clinical practice.

## Data Availability

The datasets used and/or analyzed during the current study are available from the corresponding author on reasonable request.
